# Infections in patients with aplastic Anemia in Chiang Mai University

**DOI:** 10.1186/s12878-018-0129-9

**Published:** 2018-12-04

**Authors:** Rapee Lertpongpiroon, Ekarat Rattarittamrong, Thanawat Rattanathammethee, Chatree Chai-Adisaksopha, Adisak Tantiworawit, Parichat Salee, Lalita Norasetthada

**Affiliations:** Division of Hematology, Department of Internal Medicine, Faculty of Medicine, Chiang Mai University, Chiang Mai, 50200 Thailand

**Keywords:** Infections, Aplastic anemia

## Abstract

**Background:**

Infection is a major complication in aplastic anemia (AA) patients. Primary objectives of this study were to determine the prevalence of infections and to determine types of pathogens associated with infections in patients with AA. Secondary objectives were to evaluate overall survival after infections as well as risk factors of infections in patients with AA.

**Methods:**

The authors retrospectively evaluated the infectious episodes (IEs), type of infections, associated pathogens, and outcomes of infections in patients with AA who were diagnosed and treated at Chiang Mai University between January 2010 and December 2015.

**Results:**

Sixty-seven patients with a median age of 51 years (range, 15–87 years) were enrolled. Forty two patients (62.6%) were severe AA. Median absolute neutrophil count (ANC) was 984 /mm^3^ (range, 120–5500/mm^3^). Twenty five patients (37.3%) received antithymocyte globulin plus cyclosporine A, 41 patients (61.1%) received anabolic hormone, and 2 patients (2.9%) underwent allogeneic hematopoietic stem cell transplantation. Overall, 31 IEs were documented in 22 patients (32.8%). The most common microbiologically documented site of infection was bloodstream infection (23.4%) followed by pulmonary infection (14.9%). Culture-negative febrile neutropenia occurred in 12.7%. Common pathogens identified were bacteria (73.9%), mainly gram-negative (52.9%) including *Acinetobacter baumannii* (23.5%) and *Pseudomonas aeruginosa* (17.6%)*.* Fungal infections were diagnosed in 21.7% and all were *Aspergillus spp.* Six patients (9%) died during the study period. All of them died from infection which gram-negative bacteria were most common pathogens (66.7%). Patients with infections had 5-year overall survival of 72% that is significantly less than patients without infection (100%) (*p* = 0.0002). Only risk factor that correlates with high probability of infection was ANC < 500/mm^3^. (HR 2.29, 95%CI 1.03–7.72, *p* = 0.043).

**Conclusions:**

Prevalence of infections in AA patients in Chiang Mai University was 32.8% Bacterial infections especially gram-negative bacteria were the major pathogens. Patients with ANC < 500/mm^3^ had higher risk of infections. Infection was the most important cause of death in AA.

## Background

Aplastic anemia (AA) is characterized by an empty or hypocellular bone marrow that leads to hematopoietic failure and pancytopenia [1, 2]. The immune-mediated destruction of hematopoietic stem cells by T-lymphocytes is implicated in the pathogenesis [1]. In addition, AA may relate to environmental exposures including chemicals, medical, and infectious agents as well as individual host factors [1–3]. The disease occurs more frequently in Asia than in the West, with higher incidence rates for 2- to 3-fold [3]. In Thailand, the incidence of AA appears to vary geographically. There were 3.9 cases per million persons in Bangkok, 3.0 per million in Songkla, and 5.0 per million in Khonkaen [4].

Infections are major causes of morbidity and mortality in this population as a consequence of neutropenia [1, 2, 5, 6]. The study from M.D.Anderson Cancer Center showed 41% of AA patients died from infection [5]. In that study, bacterial infection, mainly gram-positive cocci was the most common organism (55%) [4, 5]. However, fungal infections were significant causes of death [5, 6]. Over the past two decades, the development of effective treatments of AA including immunosuppressive therapy with antithymocyte globulin plus cyclosporine (ATG-CsA) and allogeneic hematopoietic stem cell transplantation (HSCT) [1, 2] as well as advanced in anti-infective therapies led to striking decreased in infection-related mortality [7].

Since there was no previous study about characteristics of infections in patients with AA in Chiang Mai University. Therefore, we evaluated the prevalence of infection, associated pathogens, and outcomes of infectious episodes (IEs) in patients with AA who were treated at Chiang Mai University.

## Methods

### Study objectives

Primary objectives of this study were to determine the prevalence of infections and to determine types of pathogens associated with infections in patients with AA. Secondary objectives were to evaluate overall survival after infections as well as risk factors of infections in patients with AA.

### Study overview

We conducted a retrospective review of the medical records of all patients who had been diagnosed with AA and had been treated and followed up at Maharaj Nakorn Chiang Mai Hospital, Chiang Mai University, Chiang Mai, Thailand between January 2010 and December 2015. Information on patient age, gender, type of AA, complete blood count, infectious prophylaxis, and therapy were collected. Characteristics of IEs including site of infections, pathogens, and outcomes of infections were analyzed. For patients with AA who underwent allogeneic HSCT, the data about IEs was collected before the procedure. Ethical approval was obtained from Research Ethics Committee, Faculty of Medicine, Chiang Mai University. The need for informed consent has been waived according to national regulations.

### Categorization and treatment of AA

AA was categorized as severe AA and non-severe AA on the basis of the degree of cytopenia. Severe AA was defined by hypocellular bone marrow (cellularity less than 25%) and at least two of followings: (1) anemia with corrected reticulocyte count < 1% or absolute reticulocyte count < 60,000/mm^3^, (2) absolute neutrophil count (ANC) ≤ 500/mm^3^, and (3) platelet count ≤ 20,000/mm^3^. Non-severe AA was defined by hypocellular bone marrow but degree of cytopenia did not meet criteria of severe AA [2]. For treatment options, the patients were managed according to Thai Guidelines for diagnosis and management of aplastic anemia [8]. Briefly, severe AA patients who did not undergo allogeneic HSCT were treated with ATG-CsA. Non-severe AA patients and severe AA who were not suitable for ATG-CsA were treated with anabolic hormone. Antimicrobial prophylaxis including co-trimoxazole, acyclovir, fluconazole, and lamivudine were prescribed in some patients who received immunosuppressive therapy according to physician’s decision. No other antimicrobial prophylaxis for gram-negative bacteria was used in this study.

### Characterization and treatment of infectious episodes (IE)

All IEs from both out-patient and in-patient setting were reviewed from the date of diagnosis until death or the last follow-up. The site of the primary infection was recorded as one of the followings: bloodstream, pulmonary, upper respiratory tract, gastrointestinal tract, genitourinary tract, musculoskeletal, febrile neutropenia [9], or others. Severe IE was defined as IE with evidence of sepsis or septic shock according to established criteria [10]. Antibiotic selection for treatment of each IE depended on institute treatment guidelines and drug-resistance data at the time of IEs, in addition to specific risk of infection. Patients with febrile neutropenia received treatment according to the international guideline [9]. All patients with febrile neutropenia were admitted and received piperacillin-tazobactam, ceftazidime, imipenem, or meropenem as initial empirical antibiotics. Patients were considered to have been cured of the infection if the signs and symptoms of infection had been resolved.

### Statistical methods

Data were entered and analyzed using the IBM SPSS Statistics 22 software. The patient’s clinical and laboratory characteristics were described in percentage, median and range. For comparison between patients with infections and without infections, differences between categorical variables were determined by Chi-square test or Fisher exact test whereas student t-test was used to compare between non-categorical variables. Survival of AA patients with and without infections were analyzed and presented as Kaplan-Meier survival curve. Each reported analysis was considered statistically significant if *p* values ≤ 0.05.

## Results

### Clinical characteristics of patients

Sixty-seven patients with a diagnosis of AA with median age of 51 years (range, 15–87 years) were included in this analysis. Forty two patients (62.7%) had severe AA. Median ANC was 984/mm^3^ (range, 120–5500/mm^3^). Twenty five patients (37.3%) received ATG-CsA, 41 patients (61.2%) received anabolic hormone, and 2 patients (3.2%) underwent HSCT. Central venous catheter insertion was performed only in HSCT patients.

### Infectious episodes (IEs)

Twenty-two patients (32.8%) developed 31 IEs with median of 1 IEs per patient (range 1–4). Clinical characteristics of patients who had IEs and did not have IEs were demonstrated in Table 1. Median time between date of diagnosis and date of first infection is 3.3 months (range (0–55.7). Twenty-seven IEs (87.1%) occurred in 21 patients required hospitalization. Average length of hospital stay due to IEs was 9 days (range 1–99 days). The most common site of infection was bloodstream infection (23.4%) followed by pulmonary infection (14.9%). Culture-negative febrile neutropenia occurred in 12.7%. (Table 2) Twenty-five IEs (80.6%) occurred in severe neutropenia patients (ANC < 500/mm^3^) with median ANC at the onset of IEs of 245/mm^3^ (range 12–3450/mm^3^). Six IEs (27.3%) occurred in patients who received infectious prophylactic drugs. (Table 1).

**Table 1:** Clinical characteristics of aplastic anemia patients with and without infectious episodes

Characteristics	Total	Without IEs	With IEs	*p*-value
	(*N* = 67)	(*N* = 45) (67.2%)	(*N* = 22) (32.8%)	
Age (years); Median (range)	51 (15–87)	49 (15–87)	49 (18–75)	0.459
Male, N (%)	37 (55.2)	27 (60.0)	10 (45.4)	0.052
Severe Aplastic Anemia, N (%)	42 (62.8)	27 (57.45)	21 (72.41)	0.189
CBC at Diagnosis				
Hemoglobin; Median (range) (g/dl)	7.7 (2.5–12.6)	7.6 (2.5–12.6)	7.5 (2.7–12.0)	0.536
White Blood Cells; Median (range) (/mm^3^)	2995 (120–8020)	3070 (1470–5300)	1870 (70–6900)	0.287
Absolute Neutrophil Count; Median (range) (/mm^3^)	984 (120–5500)	984 (147–2418)	655 (12–3208)	0.022
Platelet; Median (range) (/mm^3^)	14,000 (1000-125,000)	17,000 (4000-104,000)	14,000 (1000-125,000)	0.275
Treatment with ATG-CsA				
Received Treatment, N (%)	25 (37.3)	14 (31.1)	11 (50.0)	0.789
Time Period of Treatment; Median (range) (months)	14.6 (0.8–38.1)	15.2 (4.1–36.2)	4.7 (0.8–38.1)	0.439
Response, N (%)	18 (70.0)	10 (71.4)	7 (63.6)	0.745
Treatment with Anabolic Hormone				
Received Treatment, N (%)	41 (61.2)	28 (62.2)	13 (59.1)	0.545
Time Period of Treatment; Median (range) (months)	10.4 (1.7–104.1)	12.5 (2.1–50.6)	10.4 (1.7–104.1)	0.850
Response, N (%)	38 (92.7)	27 (96.4)	11 (84.6)	0.071

*IEs* infectious episodes, *CBC* complete blood count, *ATG-CsA* antithymocyte globulin and cyclosporine A

**Table 2:** Site of Infections and Pathogen in Aplastic Anemia Patients

Site of Infections	Blood-Stream	Pulmonary	GI Tract	GU Tract	UR Tract	Musculo-skeletal	Other Organs	Febrile Neutro-penia	Total Infections in 31 IEs*
Bacteria *Acinetobacter baumannii* *Pseudomonas aeruginosa* *Bacillus cereus* *Bacillus* spp. *Klebsiella pneumonia ESBL* Coagulase-negative *Staphylococcus* *Enterococcus fecalis* *Enterococcus faecium*	10 3 2 2 1 0 0 1 1	3 1 1 0 0 0 0 0 1	2 0 0 1 0 1 0 0 0	2 0 0 0 0 1 1 0 0	0 0 0 0 0 0 0 0 0	0 0 0 0 0 0 0 0 0	0 0 0 0 0 0 0 0 0	0 0 0 0 0 0 0 0 0	17/47 (36.1%) 4 3 3 1 2 1 1 2
Fungi *Aspergillus* spp.	1 1	4 4	0 0	0 0	0 0	0 0	0 0	0 0	5/47 (10.6%) 5
Parasite *Strongyloides stercoralis*	0 0	0 0	1 1	0 0	0 0	0 0	0 0	0 0	1 (2.1%) 1
Culture-Negative	0	0	3	2	4	3	6	6	24 (51.1%)
Total (%)	11 (23.4%)	7 (14.9%)	6 (12.7%)	4 (8.5%)	4 (8.5%)	3 (6.4%)	6 (12.7%)	6 (12.7%)	47 (100%)

*GI* gastrointestinal, *GU* genitourinary, *UR* upper respiratory, *IEs* infectious episodes, *ESBL* extended spectrum beta-lactamase

### Pathogens

There were 47 infections from 31 IEs since some IEs had co-infections (Table 2). The identifiable pathogens were 48.9% of all infections. The majority of identified pathogens of IEs were bacterial infections (73.9%), followed by fungal infections (21.7%) and parasitic infestations (4.3%). Gram negative bacteria accounted for 52.9% of all bacterial infections. *Acenitobacter baumanii* were the most frequently isolated pathogens (23.5%), followed by *Pseudomonas aeruginosa* (17.6%) and *Bacillus cereus* (17.6%). Aspergillus species were the only fungal infection that documented in this study. Only one IE with Aspergillus was proven invasive fungal disease due to positive blood culture concurrent with pulmonary infection. Other 3 IEs with Aspergillus species were probable invasive fungal diseases according to EORTC criteria [11]. *Strongyloides stercoralis* found in stool was the only parasitic infestation (2%). Eleven IEs (35.5%) occurred in patients who received ATG-CsA treatment. Most common infections in this subgroup were gram-negative bacilli (54.5%) followed by *Bacillus spp*. (27.2%).

### Risk factors of infections

In univariable analysis, variables that significantly associated with IEs were severe AA and severe neutropenia (ANC ≤ 500/mm^3^) at the time of IEs. Treatment with ATG-CsA did not statistically significant associated with IEs (RR 2.152, 95%CI 0.892–5.193, *p* = 0.081). Final model from Cox regression analysis showed severe neutropenia was independent risk factors of IEs [RR 2.83 (95%CI 1.03–7.72), *p* = 0.043] (Table 3).

**Table 3:** Risk factors of infection in patients with aplastic anemia from univariable analysis and multivariable analysis

Characteristics	Univariable Analysis		Multivariable Analysis	
	RR (95% CI)	*p*-value	RR (95% CI)	p-value
Severe Neutropenia (ANC < 500/mm^3^)	4.39 (1.82–10.60)	0.0003	2.83 (1.03–7.72)	0.043
Severe Aplastic Anemia	5.49 (1.61–18.73)	0.002	1.45 (0.60–3.50)	0.406
Previous Use of ATG-CsA	2.15 (0.89–5.19)	0.081	2.19 (0.91–5.73)	0.074

*RR* relative risk, *95%CI* 95% confidential interval, *ANC* absolute neutrophil count, *ATG-CsA* antithymocyte globulin and cyclosporine A

### Characteristics of IEs related to severity of neutropenia

**Fig. 1:**
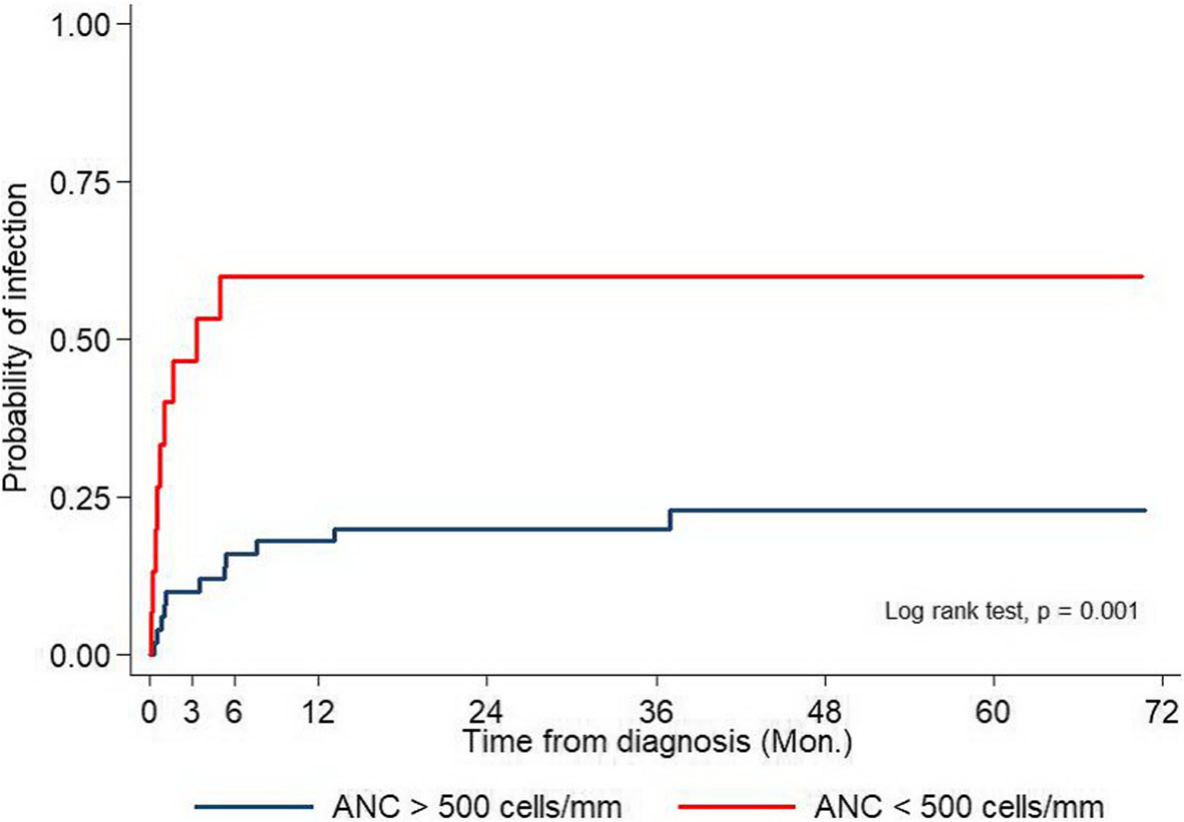
Probability of infection according to severity of neutropenia. Blue line indicates patients with ANC at the time of infection more than 500 cells/mm^3^. Red line indicates patients with ANC at the time of infection less than or equal to 500 cells/mm^3^

The characteristics of IEs in patients with and without severe neutropenia are shown in Table 4. Most IEs that occurs in patients with non-severe neutropenia were culture-negative (71%). No fungal infections were observed in this group of patient. Patients with severe neutropenia had more severe IEs (*P* = 0.043) and a higher mortality rate attributed to infection (35% vs. 0%; *P* = 0.013). Patients who had severe neutropenia developed the first IEs at a median duration of 32 days (range, 0–152 days) compare to 124 days (range, 0–1110 days) in patients with non-severe neutropenia (*P* = 0.001) (Fig. 1). Severe neutropenia patients tended to have a higher incidence of documented bacterial infection (*p* = 0.138) and fungal infection (*p* = 0.113) but not statistically significant.

**Table 4:** Characteristics of infectious episodes in patients with aplastic anemia based on the degree of neutropenia

Characteristics	Severe Neutropenia (ANC < 500/mm^3^) (*N* = 17)	Non-severe Neutropenia (ANC > 500/mm^3^) (*N* = 14)	*p*-value
Type of pathogen			
Bacteria	8 (47.1%)	3 (21.4%)	0.138
Fungi	5 (29.4%)	0 (0%)	0.113
Culture-negative	5 (29.4%)	10 (71.4%)	0.020
Outcome			
Resolve	11 (64.7%)	14 (100%)	0.03
Death	6 (35.3%)	0 (0%)	0.013

*ANC* absolute neutrophil count

### Survival outcomes

Six patients (9%) died during the study period. All of them died from infections. Most of them died from bacterial infection (*n* = 5, 83.3%). Gram-negative bacteria were most common pathogens (66.7%) with *Acinetobacter baumannii* was observed in 2 patients (33.3%). Other bacterial infections included *Enterococcus fecalis* (2 patients with 1 patient had co-infection with *A. baumannii*), *Bacillus* spp. (1 patient), and *Pseudomonas aeruginosa* (1 patient). Only one patient (16.7%) died from fungal infection (*Aspergillus fumigatus).*

**Fig. 2:**
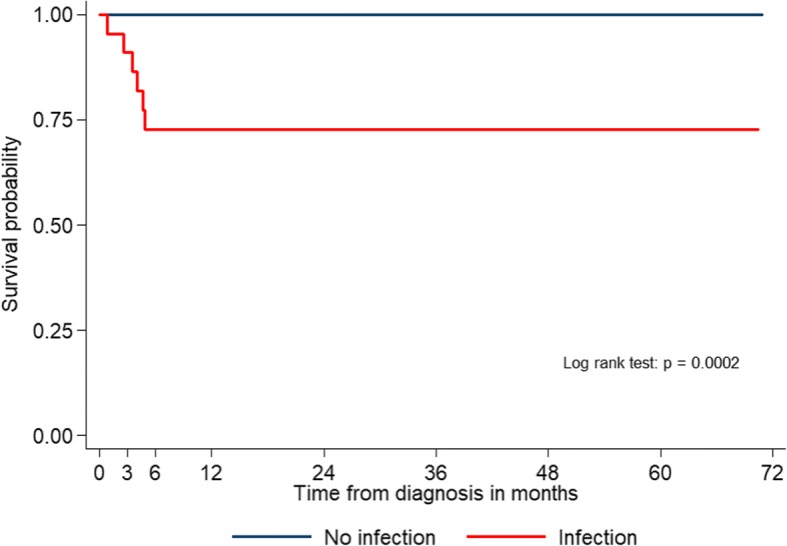
Kaplan-Meier survival curve for aplastic anemia patients according to presence of infections. Blue line indicates patients who had no infection. Red line indicates patients who had infection

Four of six patients (66.7%) had severe neutropenia. Mean ANC of patients who died from infection was 149/mm^3^ and median duration of neutropenia was 17 days. A Kaplan-Meier curve of overall survival for AA patients with and without IEs was shown in Fig. 2. Patients with infections had 5-year overall survival of 72% that is significantly less than patients without infection (100%) (*p* = 0.0002). The median survival time were not reached in both groups.

## Discussion

Infection is major complication and lead to morbidity and mortality in AA patients [1, 2, 5, 6]. The current study that included severe AA of approximately two-third of the patients showed the prevalence of infection was 32.8%. The previous study in years 1994–2000 from M.D.Anderson Cancer Center revealed the high prevalence of IEs of 81% (42 from 52 patients with 104 IEs) although proportion of severe AA was about half of patients [5]. In the studies that included only AA patients who received immunosuppressive therapy, the prevalence of infections were 68.6% in study during 1978–1989 [6] and 75–86.6% in the trial during 2005–2010 [12]. The relatively low prevalence of infection in the current study might be explained by difference in time of studies, proportion of patients with severe AA, patients’ comorbidities, as well as treatment modalities and infectious prophylaxis. It should be noted that only about one-third of patients in the current study received ATG-CsA while the majority of them were treated with anabolic hormone.

Treatment with immunosuppressive therapy with ATG-CsA is effective in patients with severe AA with response rate of 50–70% [13, 14]. However ATG-CsA suppresses T-cell function and may increase risk of infections [15]. In the current study, eleven IEs occurred in 23 patients who were receiving ATG-CsA. As a result, the prevalence of infections in this subgroup of patients was 47.8% that was still lower than prior mentioned studies [6, 12]. These findings might be partially explained by selection bias to give this treatment to young and fit patients in Thailand due to economic concern. The treatment with ATG-CsA was associated with a trend toward greater risk for infection but it is not clearly demonstrated in this study (RR 2.152, 95%CI 0.892–5.193, *p* = 0.081). This might be due to immunosuppressive effect from ATG-CsA or severe neutropenia since all patients who received this treatment must have severe AA. The authors excluded IEs that occurred in AA patients who underwent HSCT because IEs that occur in this group were also influenced by HSCT-related complications [15].

The current study confirmed that the important risk factor of infection in patients with severe AA is severe neutropenia [5–7]. Approximate 80% of IEs in this study occurred in patients with ANC < 500/mm^3^. Moreover, severe neutropenia was also associated with severe IEs, shorter duration from diagnosis to IEs, and higher mortality rate with mean ANC of the patients who died during this study was 149/mm^3^. Other risk factors that were reported to associate with infections included low absolute monocyte count and the presence of an indwelling central venous catheter [6].

According to pathogen that caused infections in AA patients, the pattern of bacterial infections in United States was changed over the last three decades [7]. First of all, there was increased prevalence of gram-positive bacteria. For instance, gram positive bacteria, especially gram-positive cocci, accounted for 76% of all bacterial infections in study from M.D.Anderson Cancer Center from 1994 to 2000 [5]. Secondly, there was decreased prevalence of infections due to coagulase-negative *Staphylococcus* species (from 53 to 25%) whereas increased prevalence of infections due to gram-positive bacilli (from 2.3 to 15%), and no significant change in the prevalence of infections due to gram-negative bacilli (from 46 to 41%) [7]. This finding was comparable with the current study since the prevalence of gram-positive bacteria (47.1%) was slightly less than gram-negative bacteria (52.9%) and gram-positive bacilli especially *Bacillus* spp. was the most common gram-positive bacteria. The low proportion of patients had central line in this study might influence the relatively low incidence of gram-positive bacterial infections. It is noteworthy that nosocomial pathogens such as *Acinetobacter baumannii* and *Pseudomonas aeruginosa* were the most common gram-negative bacterium identified. *Acinetobacter baumannii* is an emerging organism in patients with health care associated infections that has significant morbidity and mortality rates [16]. Bacterial infection caused by *Acinetobacter baumannii* was observed in one-third of patients who died in this study. Consideration of antibacterial prophylaxis against gram-negative bacilli especially fluoroquinolone was recommended in patients who expected to experience profound neutropenia for more than 7 days after chemotherapy [17]. However use antibacterial prophylaxis in AA should be based on the local policy according to the British Society for Standards in Haematology guideline [18]. According to the organisms found in this study, fluoroquinolone is probably not the effective antibiotic prophylaxis for AA patients.

Invasive fungal infections were also common pathogens in patients with AA with *Aspergillus spp.* and *Candida spp*. were the most frequently fungal infection found [5–7]. The prevalence of fungal infection in the previous studies were around 18–23% and the proportion of *Aspergillus spp.* to *Candida spp*. infections were varied from 0.85–1.57 [5, 6]. The current study showed that 21.7% of microbiological documented IEs caused by fungal infection with *Aspergillus spp.* but no *Candida spp*. was identified. It might be due to variation of degree and duration of neutropenia, immunosuppressive treatment together with types and proportion of patients who receive antifungal prophylaxis.

The significant cause of mortality of AA patients was described to be from infections. In the current study, patients with infections had significantly shorter survival than patients without infection. All patients (100%) who died in this study were resulted from infection. This proportion was higher than previous studies that reported around 40–60% [5, 6]. Overall, the infection-related mortality in AA patients in the current study was 9% that was closed to data from United States (11%) [7]. The finding that the majority of patients died from bacterial infection whereas only 16.7% died from fungal infection was correlate with decreased prevalence of invasive fungal infections in AA patients [7].

The main limitation of this study was the retrospective design that might result in incomplete data gathering including a media follow-up of all patients. The prevalence of some infections especially viral infection that required advanced laboratory testing was also lacking. The enrolled patients in this study had variation in severity as well as treatment that might lead to difficulty in data interpretation. Finally, the effect of some variables such as very severe AA (ANC < 200/mm^3^) and antimicrobial prophylaxis to IEs were not analyzed.

## Conclusions

Infections occurred in 32.8% in patients with AA in Chiang Mai University. Bacterial infections, especially gram-negative bacilli were the major cause of IEs in patients with AA as well as the most common cause of death. Prevention of infection including infectious control policy*,* early detection and early treatment of infections, and development of antibiotic guideline according to institution-based epidemiology data will lead to effective strategies to against infections in patients with AA.

## Abbreviations

AA: Aplastic anemia
ANC: Absolute neutrophil count
ATG-CsA: Antithymocyte globulin plus cyclosporine (ATG-CsA)
CBC: Complete blood count
HSCT: Hematopoietic stem cell transplantation
IEs: Infectious episodes
